# Supplementary material for: Development of a high-productivity, halophilic, thermotolerant microalga Picochlorum renovo
Source: Commun Biol. 2019 Oct 23;2:388. doi: 10.1038/s42003-019-0620-2 (PMC6811619; doi:10.1038/s42003-019-0620-2)
Supplement: Supplementary file 1 — Supplementary Information [file 42003_2019_620_MOESM1_ESM.pdf]

Supplementary Figures

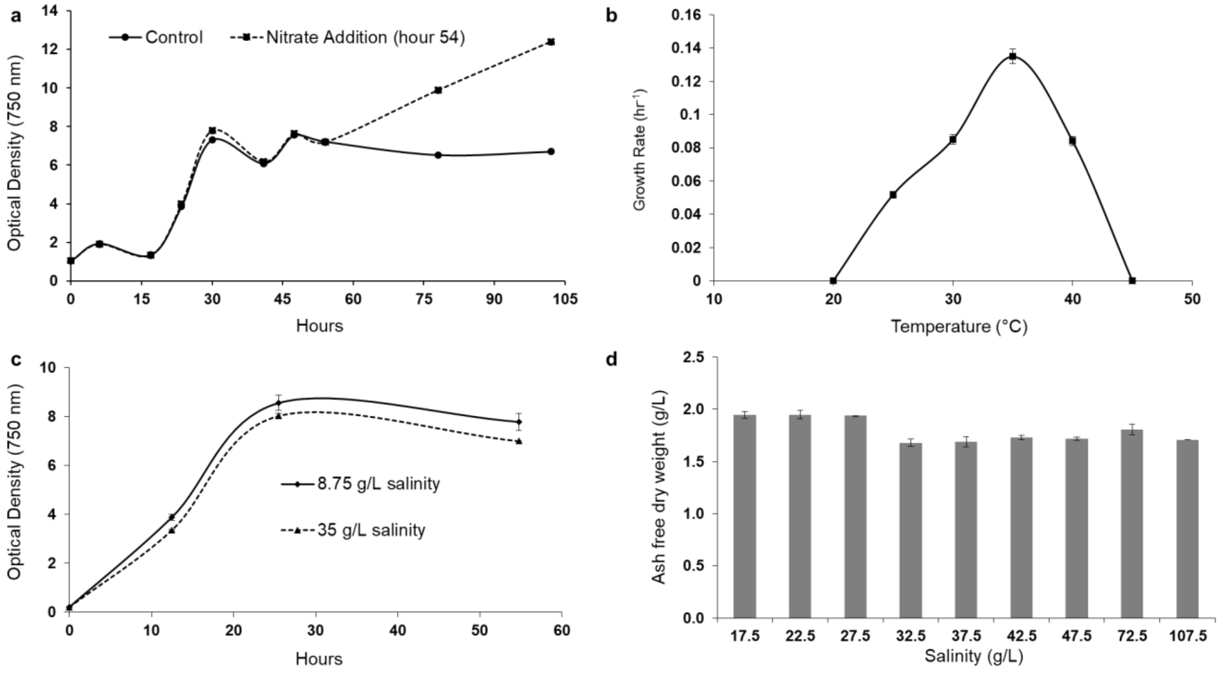

Supplementary Figure 1. Physiological characterizations under varied nitrogen, temperature, and salinity regimes. (a) Representative growth curves of *P. renovo* with and without addition of sodium nitrate to a final concentration of 4.5mM at hour 54, following entry into stationary growth phase. (b) *P. renovo* growth rate as a function of temperature. Average and standard deviation are from n=3 biological replicates. (c) Growth curve comparison of *P. renovo* at 8.75 and 35 g/L salinity. Average and standard deviation are from n=2 biological replicates. (d) Endpoint biomass titer following 6 days of growth at varying salinities for *P. renovo*. Average and standard deviation are from n=2 replicates are reported.

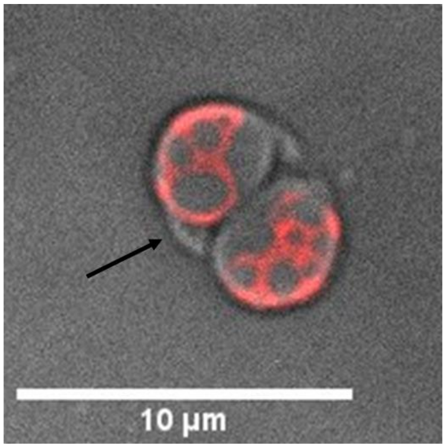

Supplementary Figure 2: Division of *P. renovo* highlighting autosporulation and mother cell wall (arrow), a defining trait of the *Picochlorum* genus. Red coloring indicates chlorophyll autofluorescence.

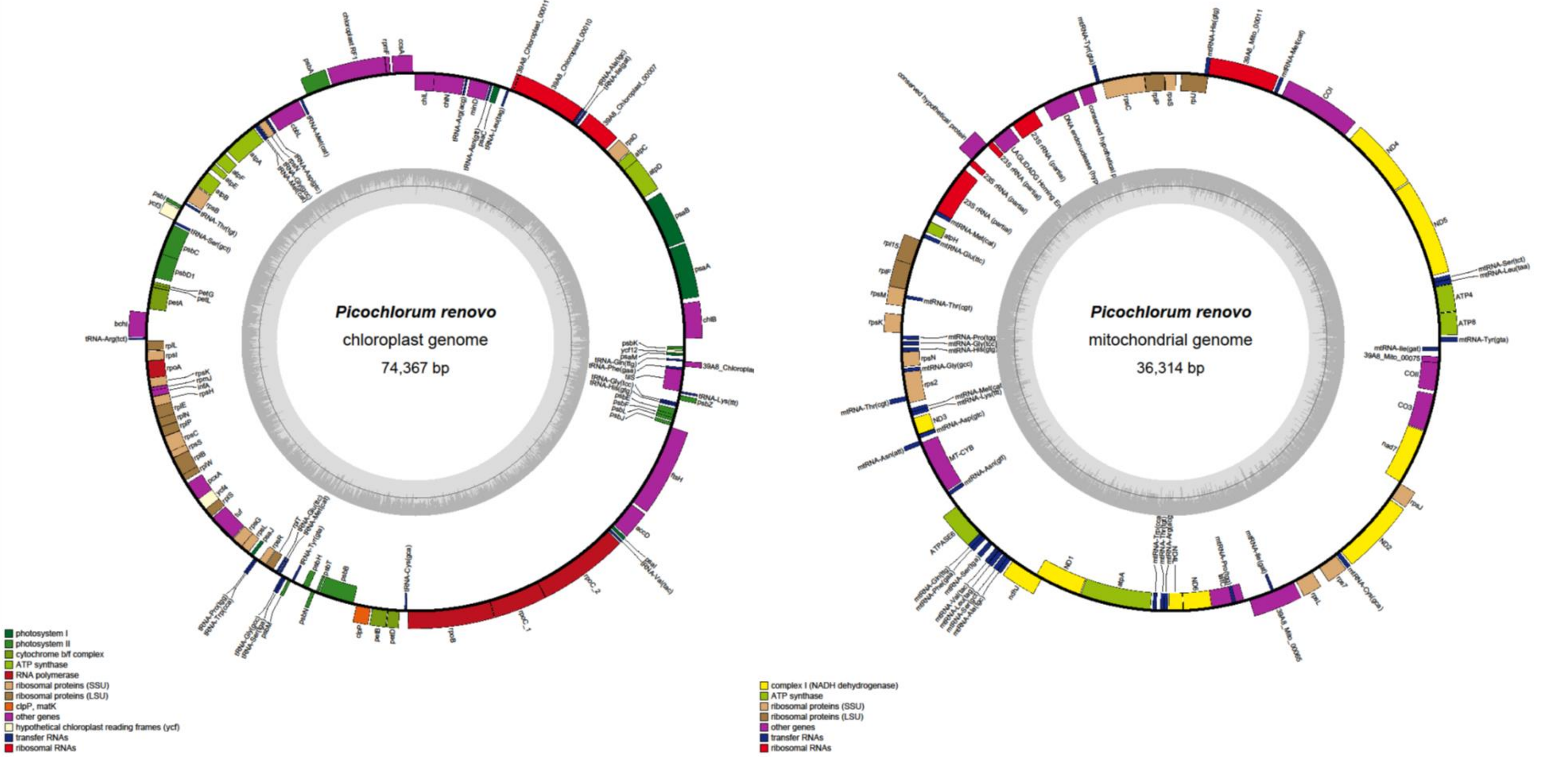

Supplementary Figure 3: Chloroplast and mitochondrial genome maps of *P. renovo*.

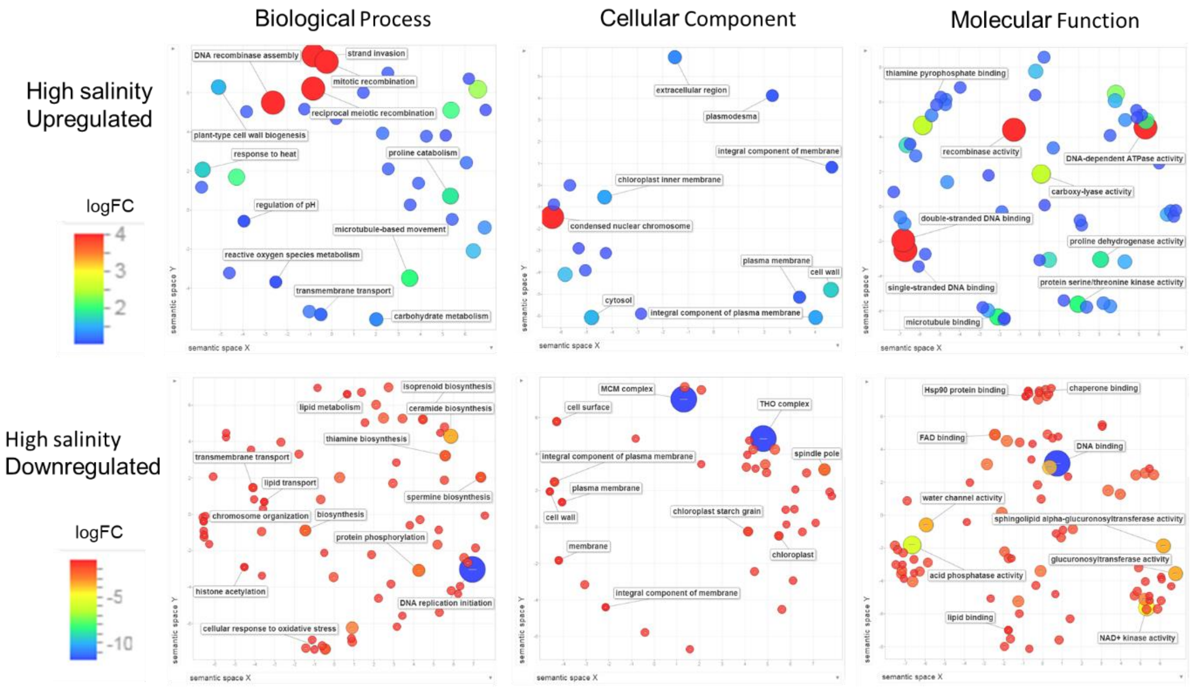

Supplementary Figure 4: **Gene ontology analysis, visualized with REVIGO**. Top panels represent gene ontology terms upregulated at high salinity, with both color and circle size indicating log fold change, as indicated in the color scale. Bottom panels represent gene ontology terms downregulated at high salinity, with both color and circle size indicating log fold change, as indicated in the color scale.

Figure 3

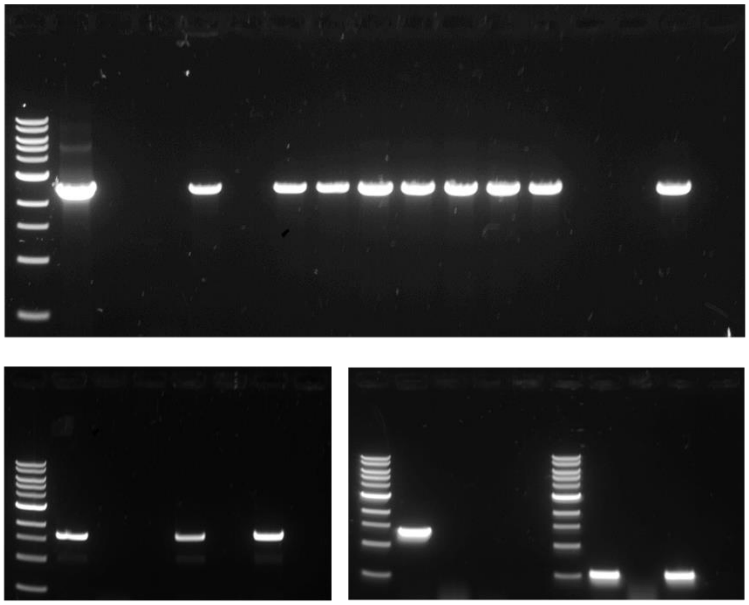

Figure 4

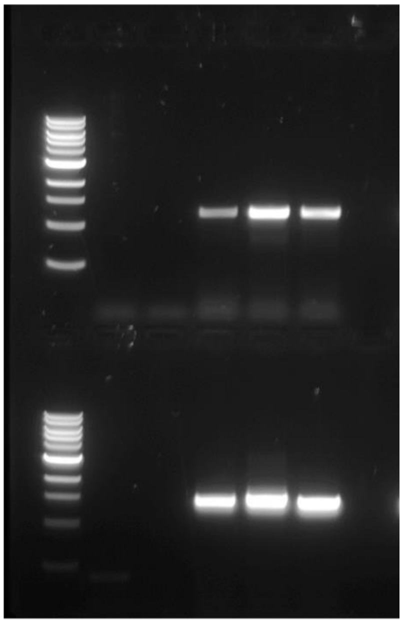

Supplementary Figure 5. Uncropped gels from Figures 3 and 4.

Supplementary Tables

Supplementary Table 1: Whole genome alignment analysis of *Picochlorum spp*.

Strain ID	Genome alignment (% identity)	Genome alignment (% query coverage)
UTEX B2795 (*Picochlorum oklahomensis*)	94	100
SENEW3 (*Picochlorum sp.*)	94	97
RCC4332 (*Picochlorum costavermella*)	87	62
NBRC102739 (*Picochlorum sp.*)	87	1.6
DOE101 (*Picochlorum soloecismus*)	88	0.7
UTEX LB 1998 (*Picochlorum oculata*)	88	0.6
