# Supplementary material for: Development of a high-productivity, halophilic, thermotolerant microalga Picochlorum renovo
Source: Commun Biol. 2019 Oct 23;2:388. doi: 10.1038/s42003-019-0620-2 (PMC6811619; doi:10.1038/s42003-019-0620-2)
Supplement: Supplementary file 2 — Description of additional supplementary items [file 42003_2019_620_MOESM2_ESM.pdf]

Supplementary Data File 1 (supplied as an excel file): All differentially expressed genes from mid log phase *P. renovo* cultures grown at 8.75 and 35 g per L seawater salinity.

Supplementary Data File 2 (supplied as an excel file): DNA elements: promoters, terminators, introns, selection markers, reporters, and primers utilized in this study. Transformation efficiencies and mCherry fluorescence of alternative nuclear engineering constructs are also reported.

Supplementary Data File 3 (supplied as an excel file): Source data underlying the graphs and charts presented in the main figures.
