# Supplementary material for: Development of a high-productivity, halophilic, thermotolerant microalga Picochlorum renovo
Source: Commun Biol. 2019 Oct 23;2:388. doi: 10.1038/s42003-019-0620-2 (PMC6811619; doi:10.1038/s42003-019-0620-2)
Supplement: Supplementary file 6 — Reporting Summary [file 42003_2019_620_MOESM6_ESM.pdf]

nature research

Corresponding author(s): Michael T. Guarnieri

Last updated by author(s): Aug 22, 2019

Reporting Summary

Nature Research wishes to improve the reproducibility of the work that we publish. This form provides structure for consistency and transparency in reporting. For further information on Nature Research policies, see Authors & Referees and the Editorial Policy Checklist .

Statistics

For all statistical analyses, confirm that the following items are present in the figure legend, table legend, main text, or Methods section.

n/a	Confirmed	
☐	☒	The exact sample size (n) for each experimental group/condition, given as a discrete number and unit of measurement
☐	☒	A statement on whether measurements were taken from distinct samples or whether the same sample was measured repeatedly
☐	☒	The statistical test(s) used AND whether they are one- or two-sided<br>*Only common tests should be described solely by name; describe more complex techniques in the Methods section.*
☐	☒	A description of all covariates tested
☐	☒	A description of any assumptions or corrections, such as tests of normality and adjustment for multiple comparisons
☐	☒	A full description of the statistical parameters including central tendency (e.g. means) or other basic estimates (e.g. regression coefficient) AND variation (e.g. standard deviation) or associated estimates of uncertainty (e.g. confidence intervals)
☐	☒	For null hypothesis testing, the test statistic (e.g. F, t, r) with confidence intervals, effect sizes, degrees of freedom and P value noted<br>*Give P values as exact values whenever suitable.*
☒	☐	For Bayesian analysis, information on the choice of priors and Markov chain Monte Carlo settings
☒	☐	For hierarchical and complex designs, identification of the appropriate level for tests and full reporting of outcomes
☒	☐	Estimates of effect sizes (e.g. Cohen's d, Pearson's r), indicating how they were calculated

Our web collection on statistics for biologists contains articles on many of the points above.

Software and code

Policy information about availability of computer code

Data collection	No proprietary software or computer code was employed in this study.
Data analysis	Data analysis was performed in Microsoft Excel (version 1905), HTStream (version 0.1.1), Salmon (version 0.13.1), R (version 3.6.0), edgeR (version 3.26.4), NUCleotide MUMmer (version 3.1), Revigo (web server), FALCON (version 0.2.2), BRAKER (version 2)

For manuscripts utilizing custom algorithms or software that are central to the research but not yet described in published literature, software must be made available to editors/reviewers. We strongly encourage code deposition in a community repository (e.g. GitHub). See the Nature Research guidelines for submitting code & software for further information.

Data

Policy information about availability of data

All manuscripts must include a data availability statement. This statement should provide the following information, where applicable:

- Accession codes, unique identifiers, or web links for publicly available datasets
- A list of figures that have associated raw data
- A description of any restrictions on data availability

The algal strain and raw data supporting the conclusions of this manuscript will be made available by the authors, without undue reservation, to any qualified researcher. The genome for Picochlorum renovo is publicly available at https://greenhouse.lanl.gov/greenhouse/. The raw data supporting the conclusions of salinity transcriptomics is available from the Sequence Read Archive, under submission number SUB5895590.

Field-specific reporting

Please select the one below that is the best fit for your research. If you are not sure, read the appropriate sections before making your selection.

Life sciences study design

All studies must disclose on these points even when the disclosure is negative.

Sample size	Sample size was determined based on the research team's prior experience utilizing the described methods. These samples sizes and methods have provided a history of peer-reviewed, statistically significant data.
Data exclusions	No data were excluded from the analyses.
Replication	All attempts to reproduce the data have been successful, biological replication was used to test for reproducibility.
Randomization	Randomization was not applicable to our study, as stochastic effects are not to be expected given our methods.
Blinding	Blinding was not applicable to our study, as the methods utilized for data collection and interpretation are mathematically derived and not subject to the placebo or observer effect.

Reporting for specific materials, systems and methods

We require information from authors about some types of materials, experimental systems and methods used in many studies. Here, indicate whether each material, system or method listed is relevant to your study. If you are not sure if a list item applies to your research, read the appropriate section before selecting a response.

Materials & experimental systems

n/a	Involved in the study
☒	☐ Antibodies
☐	☒ Eukaryotic cell lines
☒	☐ Palaeontology
☒	☐ Animals and other organisms
☒	☐ Human research participants
☒	☐ Clinical data

Methods

n/a	Involved in the study
☒	☐ ChIP-seq
☒	☐ Flow cytometry
☒	☐ MRI-based neuroimaging

Eukaryotic cell lines

Policy information about cell lines

Cell line source(s)	The source of the described cell line is from an algal culture collection, described in the following publication: Elliott et al. Algal Research 1 (2012) 102–113
Authentication	The cell line described in the manuscript is a novel isolate from the environment.
Mycoplasma contamination	Cell lines were not tested for Mycoplasma.
Commonly misidentified lines (See ICLAC register)	No commonly misidentified lines were utilized
